# Enhancing fermentation quality and fiber decomposition of *Phragmites australis* silage by introducing *Bacillus subtilis* and lactic acid bacteria consortia

**DOI:** 10.3389/fvets.2025.1557614

**Published:** 2025-05-21

**Authors:** Yong Liu, Songbai Zhang, Junrui Liao, Nazir Ahmad Khan, Shaoxun Tang, Chuanshe Zhou, Zhiliang Tan, Asmaa Elnagar, Ibrahim F. Rehan, František Zigo, Abdelfattah Z. M. Salem

**Affiliations:** ^1^Key Laboratory of Forage Breeding-by-Design and Utilization, Key Laboratory for Agro-Ecological Processes in Subtropical Region, National Engineering Laboratory for Pollution Control and Waste Utilization in Livestock and Poultry Production, and Hunan Provincial Key Laboratory of Animal Nutritional Physiology and Metabolic Process, Institute of Subtropical Agriculture, the Chinese Academy of Sciences, Changsha, China; ^2^Liuyang Liu'an Agricultural Technology Comprehensive Development Co., Ltd., Liuyang, China; ^3^Department of Pathobiochemistry, Faculty of Pharmacy, Meijo University, Nagoya, Japan; ^4^Department of Husbandry and Development of Animal Wealth, Faculty of Veterinary Medicine, Menoufia University, Shebin Alkom, Egypt; ^5^Department of Animal Nutrition and Husbandry, University of Veterinary Medicine and Pharmacy, Košice, Slovakia; ^6^Facultad de Medicina Veterinaria y Zootecnia, Universidad Autónoma del Estado de México, Toluca, Mexico; ^7^Dipartimento di Scienze del Suolo, della Pianta e degli Alimenti (Di.S.S.P.A.), Università degli Studi di Bari, Bari, Italy

**Keywords:** silage, microbial inoculant, homofermentative, heterofermentative, *Lactobacillus*, reed, silage quality

## Abstract

**Introduction:**

As a low-cost, high-fibre biomass resource, *Phragmites australis* (reed) has significant potential for feed applications, particularly as a partial replacement for conventional roughage in ruminant diets.

**Methods:**

This study investigated the effects of integrating *Bacillus subtilis* (*B. subtilis* BNCC109047) with homofermentative/ heterofermentative lactic acid bacteria (LAB) consortia on the fermentation and nutritional quality of *Phragmites australis* (reed) silage. Five treatments were evaluated: a Control (CK, without inoculum) and four inoculants—LAB (1.5 × 108 CFU/kg LAB, 1:4 homofermentative (*Lentilactobacillus plantarum* BNCC 336421 and *Pediococcus pentosaceus* BNCC 135034 in a ratio of 1:1): heterofermentative (*L. buchneri* BNCC 187961) ratio), LAB-BS2.5 (LAB plus 2.5 × 10^7^ CFU/kg *B. subtilis*), LAB-BS5.0 (LAB plus 5.0 × 10^7^ CFU/kg *B. subtilis*), and LAB-BS10.0 (LAB plus 1.0 × 10^8^ CFU/kg *B. subtilis*)—with triplicate samples per group. Silage fermentation was conducted for 90 days.

**Results:**

LAB-BS10.0 demonstrated superior fermentation outcomes, achieving the highest lactic acid-to-total acid ratio (62.3%, *p* < 0.05) and the lowest ammonia nitrogen (NH_3_-N) content (0.60 ± 0.09 g/kg, *p* < 0.05). Acetic and butyric acid concentrations were significantly reduced (*p* < 0.05), while neutral detergent fiber (NDF) decreased by 5.9% compared to the Control. Ether extract (EE) increased to 4.76% (*p* < 0.01), highlighting enhanced lipid preservation.

**Conclusion:**

These results emphasize the synergistic potential of *B. subtilis* and LAB to optimize *P. australis* silage, providing a sustainable strategy to enhance forage quality and tackle global feed shortages.

## Introduction

*Phragmites australis* (reed) is a perennial aquatic grass with global distribution, recognized for its high biomass yield and adaptability to wetland ecosystems (1). Its structural components—roots, stems, and leaves—have been utilized in diverse applications, ranging from construction materials to phytoremediation (2, 3). Recently, *P. australis* has gained attention as a high-biomass forage candidate, offering a sustainable alternative to conventional fodder crops (4, 5). As a promising unconventional silage material, reed shows considerable potential for forage applications. However, similar to other non-traditional feedstocks such as sorghum, barley, and oats, its use in silage systems remains poorly understood, particularly in optimizing fermentation efficiency and nutrient retention (6). The plant’s high lignocellulosic fiber content promotes undesirable microbial activity during ensiling, leading to excessive butyric acid production, pH instability, and nutrient loss (7–9). These factors compromise silage quality, limiting its adoption in livestock feed systems (10, 11).

Recent advances in silage microbiology highlight the potential of microbial inoculants to enhance fermentation quality (12, 13). Homofermentative lactic acid bacteria (LAB), such as *Lentilactobacillus plantarum* (14), excel in lactic acid (LA) production, effectively acidifying various lignocellulosic substrates, including sweet sorghum bagasse, barley, *P. australis*, corn, and rice (15). This rapid acidification process significantly suppresses spoilage microorganisms (16–18). Heterofermentative LAB, including *Lactobacillus buchneri*, play a complementary role in the silage fermentation of various forage crops, including lucerne, maize, and Napier grass. These bacteria convert residual carbohydrates to acetic acid through their unique metabolic pathway, providing an additional antimicrobial barrier against spoilage microorganisms such as molds and yeasts (19–22). However, the limited fiber-degrading capacity of LAB often hinders the utilization of lignocellulose-rich forages like *P. australis*.

To address this limitation, *Bacillus subtilis* has emerged as a promising adjunct. This bacterium produces bacteriocins that inhibit undesirable microbes while secreting cellulases and xylanases to break down recalcitrant fibers (23). Synergistically, *B. subtilis* enhances nutrient availability, elevates flavor compounds, and improves silage palatability (24–27). Despite these benefits, research on *B. subtilis*-LAB consortia in unconventional forage silage, particularly *P. australis*, remains scarce.

Current research on optimizing *P. australis* silage through microbial interventions remains sparse. While LAB is widely used to enhance silage fermentation, its synergy with fiber-degrading bacteria like *Bacillus subtilis* (*B. subtilis*) remains a mystery. This study investigates the novel combination of homofermentative/heterofermentative LAB consortia (*L. plantarum*, *Pediococcus pentosaceus*, and *L. buchneri*) with incremental doses of *B. subtilis*. We aimed to (1) evaluate the effects on fermentation parameters (e.g., LA, NH_3_-N), (2) assess fiber degradation (e.g., NDF, ADF), and (3) establish an optimal inoculant ratio for enhancing the nutritional quality and digestibility of *P. australis* silage, offering a sustainable solution to mitigate the growing feed supply–demand imbalance.

## Materials and methods

### Plant materials preparation

**Figure 1:**
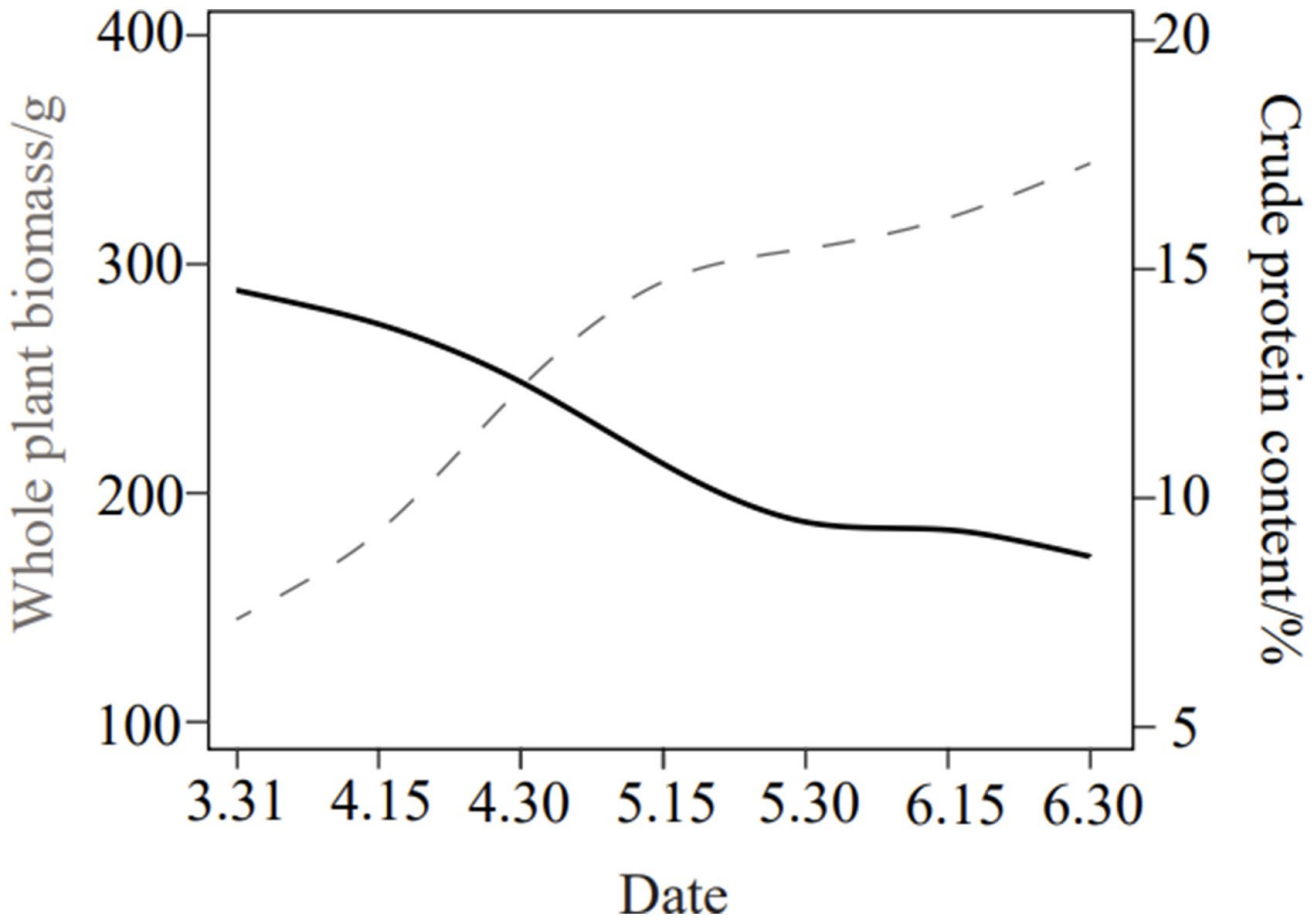
The biomass and protein content simulation among the life cycle of *P. australis*.

*Phragmites australis* (reed) was harvested at the vegetative growth stage from Dongting Lake District, Yueyang City, Hunan Province, China, in 2021. The biomass and protein content simulation of *P. australis* plant (containing stems and leaves) at the early vegetative growth stage is presented in Figure 1. The plants were chopped into 2–3 cm segments, and surface moisture was air-dried. Baseline nutrient composition of raw material was analyzed (Table 1), including dry matter (DM), crude protein (CP), water-soluble carbohydrates (WSC), neutral detergent fiber (NDF), acid detergent fiber (ADF), ether extract (EE), and gross energy (GE).

**Table 1:** Primary nutritional composition of *P. australis* (%, DM basis).

Items	Content
DM, g/kg	283.76
CP, g/kg	87.18
WSC, g/kg	21.99
NDF, g/kg	786.05
ADF, g/kg	461.87
EE, g/kg	62.26
GE, MJ/kg	16.41

### Microbial inoculants and experimental design

#### Microbial strains

Homogenenous LAB: *L. plantarum* (3.0 × 10^9^ CFU/g, BNCC 336421), *P. pentosaceus* (1.0 × 10^10^ CFU/g, NBCC 135034). Heterogeneous LAB: *L. buchneri* (1.0 × 10^10^ CFU/g, BNCC 187961). Functional bacteria: *B. subtilis* (5.0 × 10^10^ CFU/g, BNCC 109047). All strains were procured from the BeNa Culture Collection (Beijing, China).

#### Silage treatments

**Figure 2:**
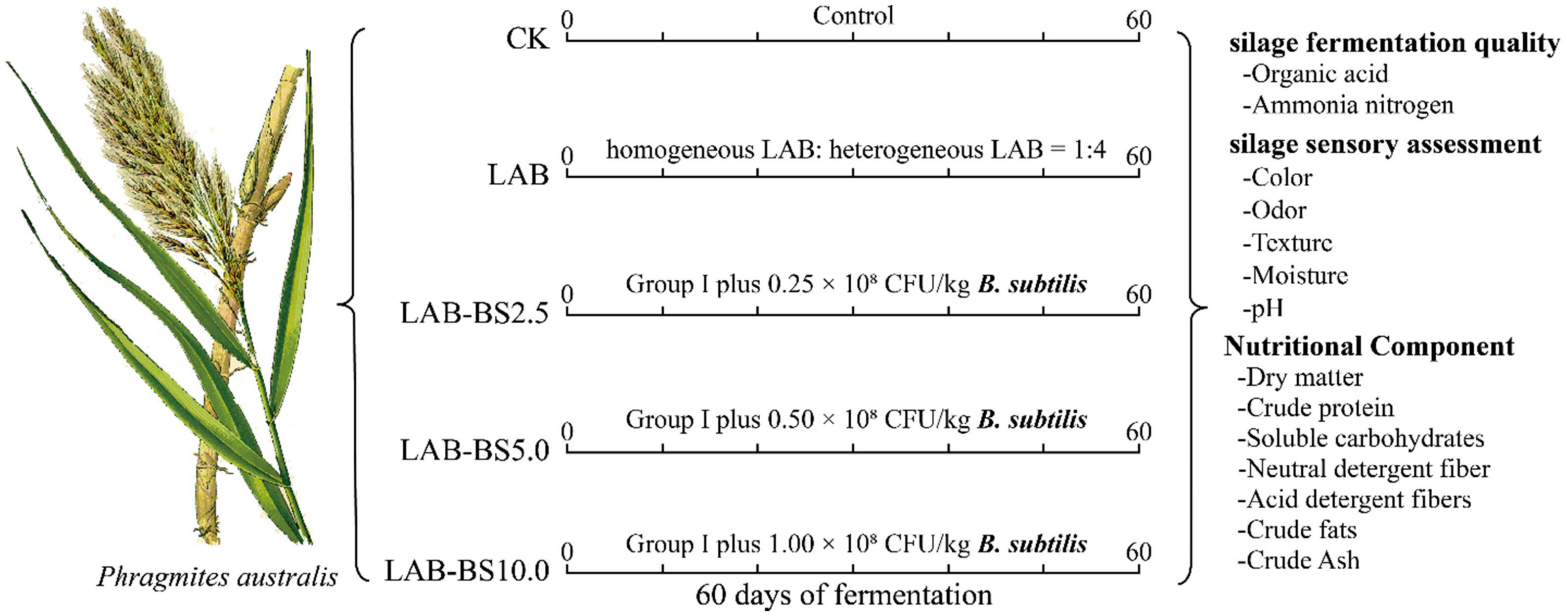
Schematic diagram of experimental design and silage processes.

Five groups with three replicates each were established, including the control group (CK), inoculum LAB, inoculum LAB-BS2.5, inoculum LAB-BS5.0, and inoculum LAB-BS10.0 groups. The control group (CK) was *P. australis* silage without inoculants. The inoculum LAB group was the *P. australis* with homogenous LAB (*L. plantarum* + *L. pentosaceus* at a 1:1 ratio) combined with heterogeneous LAB (*L. buchneri*) at a 1:4 ratio (total LAB: 1.5 × 10^8^ CFU/kg fresh silage). The inoculum LAB-BS2.5, LAB-BS5.0, and LAB-BS10.0 were LAB consortium plus *B. subtilis* with 2.5 × 10^7^, 5.0 × 10^7^, and 1.0 × 10^8^ CFU/kg, respectively. The details are presented in Table 2. The fermentation process and the determination index of silage are shown in Figure 2. The *B. subtilis* gradient (2.5 × 10^7^ to 1.0 × 10^8^ CFU/kg) was selected to evaluate dose-dependent effects on fiber degradation and fermentation efficiency.

**Table 2:** Microbial inoculant composition (CFU/kg fresh silage material) for silage.

Treatments	LAB Consortium (*L. plantarum*: *P. pentosaceus*: *L. buchneri*, in ratio of 1.0: 1.0: 8.0)	*B. subtilis* (BS)
Control (CK)	–	–
LAB	1.5 × 10^8^	–
LAB-BS2.5	1.5 × 10^8^	2.5 × 10^7^
LAB-BS5.0	1.5 × 10^8^	5.0 × 10^7^
LAB-BS10.0	1.5 × 10^8^	1.0 × 10^8^

Control (CK, without inoculum), LAB (consisting of 1.5 × 10^8^ CFU/kg of homogeneous [*L. plantarum* BNCC 336421 and *P. pentosaceus* BNCC 135034 in a ratio of 1:1], to heterogenous LAB species [*L. buchneri* BNCC 187961] in a ratio of 1:4), LAB-BS2.5 (LAB plus 2.5 × 10^7^ CFU/kg *B. subtilis*), LAB-BS5.0 (LAB plus 5.0 × 10^7^ CFU/kg *B. subtilis*), and LAB-BS10.0 (LAB plus 1.0 × 10^8^ CFU/kg *B. subtilis*).

### Silage preparation and fermentation

#### Inoculation

Microbial suspensions were prepared by dissolving strains in distilled water (10 mL/kg fresh weight), activated at 30°C for 2 h, and uniformly sprayed onto chopped *P. australis*.

#### Packing and storage

reated material was packed into 40 cm × 60 cm polyethylene bags (1 kg/bag), vacuum-sealed, and stored at 25–30°C for 90 days (18). This duration was based on preliminary trials confirming pH stabilization and organic acid equilibrium.

### Silage quality assessment

#### Silage fermentation quality

After homogenizing *P. australis* silage, 50 g of the sample was combined with 450 mL of distilled water in a sealed 500 mL Erlenmeyer flask. The mixture was refrigerated at 4°C for 24 h, filtered through polyester cloth, and pressed to extract the residual liquid. The filtrate was further purified using qualitative filter paper and stored in 15 mL centrifuge tubes for subsequent analyses.

##### pH and organic acid analysis

The pH value was measured using a calibrated pH meter (HI2211, Hanna Instruments). Lactic acid (LA) was quantified using high-performance liquid chromatography (HPLC; Agilent 1,290, USA). Volatile fatty acids, such as acetic acid (AA), butyric acid (BA), and propionic acid (PA), were analyzed using gas chromatography (GC; Agilent 7890A, USA) equipped with a flame ionization detector (FID) and an FFAP capillary column (15 m × 0.32 mm × 0.25 μm). Operational parameters included helium carrier gas (19.991 kPa), injector temperature (250°C), and a 2 μL injection volume.

##### Ammonia nitrogen (NH_3_-N) determination

NH_3_-N content was assessed using phenol-sodium hypochlorite colorimetry. Specifically, a 1.5 mL aliquot of extract was mixed with 0.15 mL of 25% metaphosphoric acid (10,1, v/v), stabilized for 30 min, and centrifuged (1,500 rpm, 4°C, 15 min). The supernatant was filtered through a 0.45 μm filter membrane prior to spectrophotometric analysis.

#### Nutritional component determination

Silage samples (200 g) were oven-dried at 65°C to constant weight, ground through a 40 mesh sieve, and stored in airtight containers. DM was determined by further drying at 105°C. Nutritional parameters were analyzed as follows: CP: Kjeldahl nitrogen method; WSC: anthraquinone-sulfuric acid colorimetry; NDF and ADF: sequential detergent filtration (Fan’s method); EE: Sohren’s extraction with petroleum ether; Crude ash: high-temperature incineration (550°C, 6 h) (24, 28).

#### Silage evaluation standards

Silage sensory quality was graded per China’s *Standard for silage quality evaluation* (Table 3), assessing: color (turquoise to dark brown, score 0–20), odor (aromatic sour to moldy, score 0–25), texture (loose to sticky, score 0–10), moisture (compacted to watery, score 0–20), and pH values (3.4–4.8, score 0–25).

**Table 3:** Silage quality evaluation standards.

Parameter	Total Score	Excellent	Good	Generally	Poor
Color	20	Turquoise/chartreuse (14~20)	Yellow-green (8~13)	Brownish-yellow (1~7)	Dark brown (0)
Odor	25	Aromatic sour (18~25)	Light sour (9~17)	Pungent sour (1~8)	Rotten/moldy (0)
Texture	10	Loose, non-sticky (8~10)	Soft, cohesionless (4~7)	Slightly viscous (1~3)	Sticky (0)
Moisture (%)	20	Moist, no droplets (14~20)	Moist, droplets (8-13)	Watery (1~7)	Drying/waterlog ged (0)
pH	25	3.4 ~ 3.8 (18 ~ 25)	3.9–4.1 (10–17)	4.2 ~ 4.7 (1 ~ 8)	> 4.8 (0)
Total Score	100	76 ~ 100	51 ~ 75	26 ~ 50	0 ~ 25
Grade		(Excellent)	(Good)	(Generally)	(Poor quality)

### Statistical analysis

All experimental data were reported as mean ± standard error (SE), indicating variability around the mean estimate. Data normality was verified using the Shapiro–Wilk test (29). One-way ANOVA with Duncan’s post-hoc test compared treatment means was conducted in SPSS (v22.0), with significance thresholds at *p* < 0.05 (significant) and *p* < 0.01 (highly significant). Spearman’s correlation coefficients were calculated using the R package psych (v 2.4.1), and results were visualized with corrplot (v 0.92).

## Results

### Sensory assessment and pH score of *P. australis* silage

All silage treatments exhibited favorable sensory profiles, with no signs of mold or spoilage (Table 4). The silage color ranged from yellow-green to brownish-yellow, displaying a soft, non-sticky texture and optimal moisture content (14–17/20) without water droplet formation. A pronounced wine-like aroma was observed across treatments, particularly in LAB-BS5.0 and the Control (CK), which scored highest in odor (12/25). pH values ranged between 3.9 and 4.1, with LAB-BS5.0 (3.98) and LAB-BS10.0 (3.94) showing lower pH than CK (3.99), while LAB-BS2.5 (4.12) and LAB-BS2.5 (4.12) and LAB (4.02) had slightly higher values. Total sensory scores classified all treatments as “Good” (60–70/100), with LAB-BS 5.0 and CK achieving the highest scores (70/100).

**Table 4:** Sensory evaluation scores of *P. australis* silage by introducing *B. subtilis* and LAB consortia.

Treatments	Index scores					Total	Grade
	Color	Smell	Structure	Moisture	pH score/values		
Control (CK)	16	12	10	17	15/3.99	70	Good
LAB	17	9	10	17	14/4.02	67	Good
LAB-BS2.5	16	10	9	16	9/4.12	60	Good
LAB-BS5.0	17	12	10	16	15/3.98	70	Good
LAB-BS10.0	15	10	10	16	16/3.94	67	Good

Control (CK, without inoculum), LAB (consisting of 1.5 × 10^8^ CFU/kg of homogeneous (*L. plantarum* BNCC 336421 and *P. pentosaceus* BNCC 135034 in a ratio of 1:1), to heterogenous LAB species (*L. buchneri* BNCC 187961) in a ratio of 1:4), LAB-BS2.5 (LAB plus 2.5 × 10^7^ CFU/kg *B. subtilis*), LAB-BS5.0 (LAB plus 5.0 × 10^7^ CFU/kg *B. subtilis*), and LAB-BS10.0 (LAB plus 1.0 × 10^8^ CFU/kg *B. subtilis*).

### Effects on organic acid profiles and ammonia nitrogen in *P. australis* silage by introducing *B. subtilis* and LAB consortia

The addition of *B. subtilis* significantly influenced organic acid profiles and NH_3_-N content (Table 5). While LAB increased LA content (1.86 ± 0.04 g/kg) compared to CK (1.76 ± 0.03 g/kg), the difference was non-significant. However, LAB-BS2.5 (2.00 ± 0.11 g/kg) and LAB-BS2.5 (1.93 ± 0.06 g/kg) showed significantly higher LA than CK (*p* < 0.05). BA and PA concentrations increased significantly in LAB (1.21 ± 0.05 g/kg and 0.43 ± 0.02 g/kg, respectively) compared to CK (*p* < 0.01). NH_3_-N decreased progressively with higher *B. subtilis* doses, reaching the lowest value in LAB-BS10.0 (0.60 ± 0.09 g/kg vs. CK: 0.89 ± 0.03 g/kg; *p* < 0.05). All treatments reduced acetic acid (AA) and PA compared to CK (*p* < 0.01), with LAB-BS10.0 showing the most pronounced reduction (AA: 2.01 ± 0.01 g/kg; PA: 0.17 ± 0.00 g/kg).

**Table 5:** Silage fermentation parameters for *P. australis* silage by introducing *B. subtilis* and LAB consortia.

Treatments	LA	AA	BA	PA	NH_3_-N
Control (CK)	1.76 ± 0.03^bc^	3.35 ± 0.01^a^	1.09 ± 0.01^b^	0.32 ± 0.00^c^	0.89 ± 0.03^a^
LAB	1.86 ± 0.04^ab^	3.41 ± 0.14^a^	1.21±0.05^a^	0.43 ± 0.02^a^	0.84 ± 0.07^a^
LAB-BS2.5	2.00 ± 0.11^a^	2.89 ± 0.15^b^	1.15 ± 0.06^ab^	0.37 ± 0.02^b^	0.79 ± 0.01^a^
LAB-BS5.0	1.93 ± 0.06^a^	3.33 ± 0.03^a^	1.14 ± 0.01^ab^	0.36 ± 0.00^bc^	0.78 ± 0.01^a^
LAB-BS10.0	1.68 ± 0.03^c^	2.01 ± 0.01^c^	0.65 ± 0.00^c^	0.17 ± 0.00^d^	0.60 ± 0.09^b^
SEM	0.04	0.18	0.07	0.03	0.03
*p*-*value*	0.02	<0.01	<0.01	<0.01	0.02

Different lowercase letters within the same column indicate significant differences between the groups at a *p*-value of 0.05. LA, lactic acid; AA, acetic acid; BA, butyric acid; PA, propanoic acid; NH_3_-N, ammonia nitrogen. Control (CK, without inoculum), LAB (consisting of 1.5× 10^8^ CFU/kg of homogeneous (*L. plantarum* BNCC 336421 and *P. pentosaceus* BNCC 135034 in a ratio of 1:1), to heterogenous LAB species (*L. buchneri* BNCC 187961) in a ratio of 1:4), LAB-BS2.5 (LAB plus 2.5 × 10^7^ CFU/kg *B. subtilis*), LAB-BS5.0 (LAB plus 5.0 × 10^7^ CFU/kg *B. subtilis*), and LAB-BS10.0 (LAB plus 1.0 × 10^8^ CFU/kg *B. subtilis*). The units (g/kg fresh silage for LA, AA, BA, PA, and NH_3_-N) represent the mass weight (g) of each parameter per kilogram of fresh silage material.

In summary, LAB-BS10.0 achieved the highest LA-to-total acid ratio (62.3%, *p* < 0.01) and the lowest NH_3_-N content, indicating superior fermentation efficiency. LAB-BS2.5 and LAB-BS5.0 demonstrated a dose-dependent enhancement in LA production, highlighting *B. subtilis*’s role in acidification.

### Effect on the nutrient content of *P. australis* silage

The inclusion of *B. subtilis* significantly influenced the nutritional profile of *P. australis* silage (Table 6). DM content decreased (*p* < 0.01) in all inoculated groups compared to CK, with the lowest values observed in LAB-BS2.5 (24.92%) and LAB-BS5.0 (24.99%). DM followed a descending order: LAB-BS10.0 (26.43%) > LAB-BS5.0 > LAB-BS2.5.

**Table 6:** Nutrient compositions of *P. australis* silage (%, DM basis) by introducing *B. subtilis* and LAB consortia.

Treatments	DM	CP	WSC	NDF	ADF	Ash	EE
Control (CK)	27.36 ± 0.01^a^	5.42 ± 0.59	0.62 ± 0.04^b^	73.70 ± 0.96^a^	26.00 ± 1.02	10.97 ± 0.02^a^	2.57 ± 0.00^c^
LAB	25.82 ± 0.06^c^	5.19 ± 0.05	0.69 ± 0.02^b^	72.42 ± 3.73^ab^	24.01 ± 1.98	9.93 ± 0.19^b^	3.10 ± 0.09^c^
LAB-BS2.5	24.92 ± 0.04^d^	5.98 ± 0.32	0.87 ± 0.02^a^	64.19 ± 2.21^c^	23.34 ± 0.65	10.40 ± 0.09^ab^	3.88 ± 0.01^b^
LAB-BS5.0	24.99 ± 0.05^d^	6.49±0.87	0.68 ± 0.05^b^	71.06 ± 1.28^ab^	24.69 ± 2.06	10.58 ± 0.55^ab^	4.62 ± 0.49^ab^
LAB-BS10.0	26.43 ± 0.02^b^	6.76 ± 0.17	0.73 ± 0.07^b^	67.80 ± 1.69^bc^	22.51 ± 1.72	8.96 ± 0.09^c^	4.76 ± 0.46^a^
SEM	0.31	0.23	0.03	1.26	0.54	0.24	0.29
*p*-*value*	<0.01	0.09	0.02	0.04	0.35	<0.01	<0.01

DM, dry matter; CP, crude protein; WSC, water-soluble carbohydrates; ADF, acid detergent fiber; NDF, neutral detergent fiber; Ash, crude Ash; EE, ether extract. Control (CK, without inoculum), LAB (consisting of 1.5× 10^8^ CFU/kg of homogeneous (*L. plantarum* BNCC 336421 and *P. pentosaceus* BNCC 135034 in a ratio of 1:1), to heterogenous LAB species (*L. buchneri* BNCC 187961) in a ratio of 1:4), LAB-BS2.5 (LAB plus 2.5 × 10^7^ CFU/kg *B. subtilis*), LAB-BS5.0 (LAB plus 5.0 × 10^7^ CFU/kg *B. subtilis*), and LAB-BS10.0 (LAB plus 1.0 × 10^8^ CFU/kg *B. subtilis*).

CP content increased incrementally with higher *B. subtilis* doses, peaking in LAB-BS10.0 (6.76%), though differences from CK (5.42%) were non-significant (*p* = 0.09). LAB-BS2.5 exhibited the highest WSC (0.87%, *p* < 0.05), a 40.3% increase over CK. NDF decreased significantly in LAB-BS2.5 (64.19%, *p* < 0.05) and LAB-BS10.0 (67.80%, *p* < 0.05), representing reductions of 9.51 and 5.9%, respectively. ADF was lowest in LAB-BS10.0 (22.51%), though differences from CK (26.00%) were non-significant. Ash content decreased (*p* < 0.01) in LAB (9.93%) and LAB-BS10.0 (8.96%). EE increased progressively with *B. subtilis* dosage, reaching 4.76% in LAB-BS10.0 (*p* < 0.01). LAB-BS10.0 optimized fiber degradation (NDF: 67.80%) while enhancing CP (6.76%) and EE (4.76%). LAB-BA2.5 maximized WSC accumulation (0.87%), critical for microbial activity during fermentation.

### Correlation analysis between nutrients and fermentation parameters by introducing *B. subtilis*

**Figure 3:**
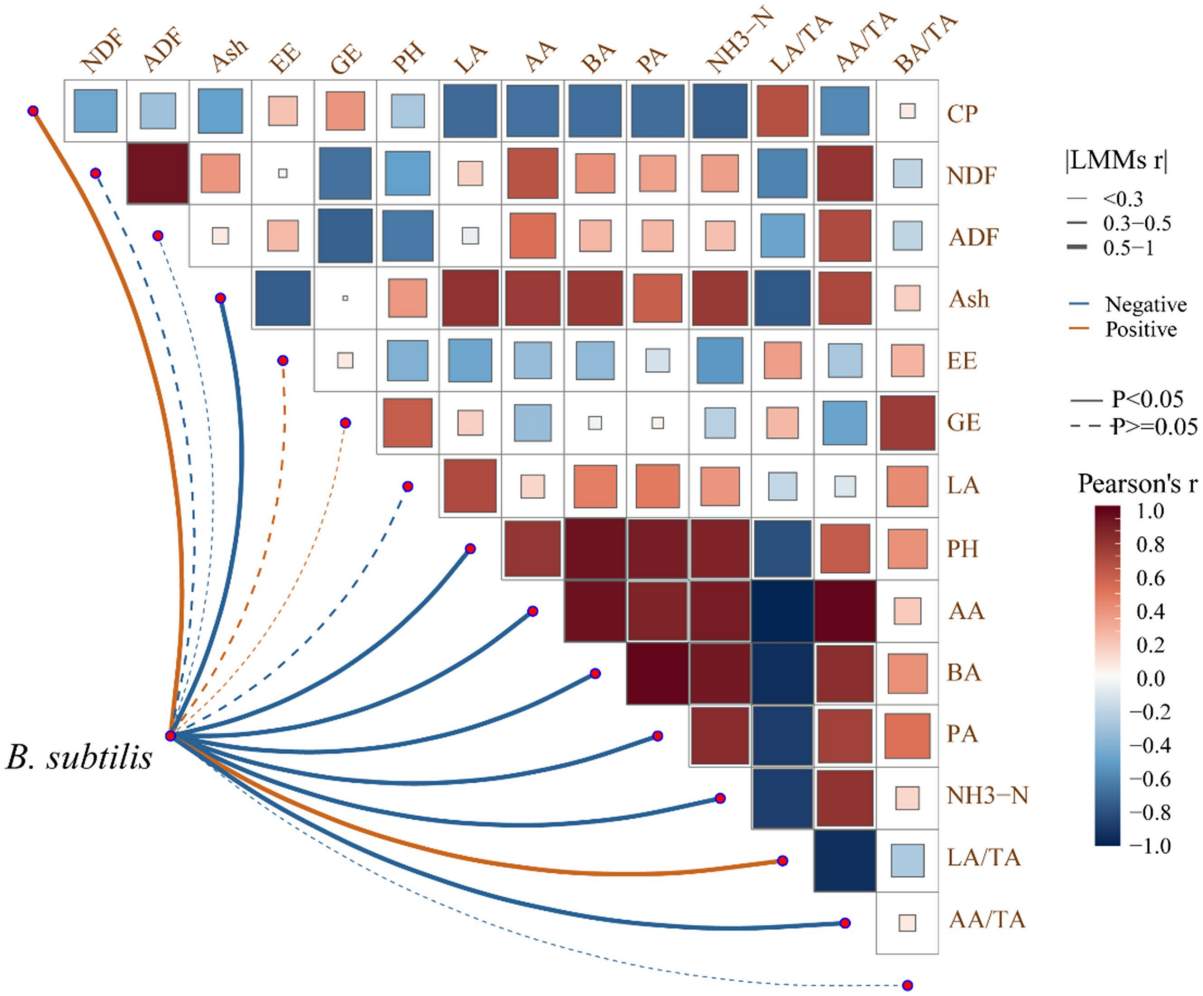
Correlation analysis between nutrients and fermentation parameters of *P. australis* by introducing *B. subtilis* in the LAB fermentation system.

Key correlations between nutrients and fermentation parameters of *P. australis* silage were identified (Figure 3). Positive correlations between NDF/ADF and AA/TA (*r* = 0.82), BA (*r* = 0.76), and NH_3_-N (*r* = 0.68) suggest that fiber-rich substrates favor acetic/butyric acid production. Positive association of *B. subtilis* with CP (*r* = 0.71) and negative correlation with NH_3_-N (*r* = −0.65) indicate its role in converting ammonia to microbial protein. *B. subtilis* correlated positively with LA/TA (*r* = 0.89) and negatively with pH (*r* = −0.78), highlighting its dual role in acid production and fiber breakdown.

*B. subtilis* enhances lactic acid dominance (LA/TA > 60%) while reducing NH_3_-N, critical for high-quality silage. Fiber degradation by *B. subtilis* improves digestibility, supporting *P. australis* as a viable unconventional forage.

## Discussion

### Effect on fermentation quality of *P. australis* silage

NH_3_-N, LA, and volatile fatty acids (VFAs) are critical indicators of silage fermentation quality (30). In this study, NH_3_-N content decreased progressively with increasing *B. subtilis* inoculation, reaching the lowest level in LAB-BS10.0 (0.60 ± 0.09 g/kg vs. Control: 0.89 ± 0.03 g/kg; *p* < 0.05). This aligns with prior findings that *B. subtilis* produces bacteriocin-like metabolites, suppressing yeasts and molds while enhancing aerobic stability and reducing NH_3_-N through proteolysis (27, 31–33). Additionally, *B. subtilis*-mediated acetolactate synthase activity likely catalyzed pyruvate conversion to acetolactic acid, improving both nutritional value and palatability (27, 32). Another factor affecting silage quality was the silage fermentation time, which ranged from 60 to 120 days, particularly 60 to 90 days (15, 18, 34).

LABs are well-documented for their role in rapid acidification, which preserves silage by inhibiting spoilage microorganisms (35–37). Homogeneous LAB (e.g., *Lentilactobacillus plantarum*) excel in LA production but offer limited inhibition of harmful bacteria (38, 39). Conversely, heterogeneous LAB (e.g., *L. buchneri*) metabolize residual sugars into AA, effectively suppressing molds (12). This study’s combination of homofermentative and heterofermentative LAB at a 1:4 ratio synergistically enhanced LA yield while maintaining AA-driven mold inhibition (Table 5).

Notably, LAB-BS10.0 achieved the highest LA-to-total acid ratio (62.3%, *p* < 0.01), despite a slight reduction in absolute LA content compared to LAB-BS2.5 (2.00 ± 0.11 g/kg). This paradox highlights *B. subtilis*’s dual role: (1) promoting fiber degradation to release fermentable substrates for LA synthesis and (2) redirecting metabolic pathways to prioritize LA over VFAs like BA and PA (40, 41). The progressive decline in AA, BA, and PA with increasing *B. subtilis* doses (*p* < 0.01) further underscores its ability to refine fermentation profiles, favoring LA dominance.

### Effect on the nutritional value of *P. australis* silage

DM content is a critical indicator of silage preservation efficiency (42). In this study, DM decreased significantly (*p* < 0.01) in all inoculated groups compared to the Control (CK: 27.36%), likely due to microbial utilization of soluble carbohydrates during fermentation (Table 6). Notably, LAB-BS20.0 retained higher DM (26.43%) than other inoculated groups, suggesting *B. subtilis* moderates substrate consumption while enhancing fiber degradation.

CP content, a key nutritional metric (43), showed no significant differences between treatments (*p* = 0.09), though LAB-BS10.0 achieved the highest CP (6.76%). This aligns with Bai et al. (44), where *B. subtilis* improved protein retention in corn silage. Conversely, Bonaldi et al. (32) observed no CP enhancement with *B. subtilis*, possibly due to differences in substrate composition.

WSC peaked in LAB-BS2.5 (0.87%, *p* < 0.05), reflecting *B. subtilis*’s role in hydrolyzing structural carbohydrates. However, higher *B. subtilis* doses reduced WSC, likely due to accelerated microbial metabolism. *B. subtilis*’s cellulase activity (45) likely contributed to NDF reduction in LAB-BS2.5 (64.19%, *p* < 0.05) and LAB-BS10.0 (67.80%, *p* < 0.05), contrasting with Guo et al. (41), who reported minimal fiber impact.

Ash content decreased significantly (*p* < 0.01) in LAB (9.93%) and LAB+BS10.0 (8.96%), likely due to BS-driven mineral solubilization. Ether extract (EE) increased progressively with BS dosage, peaking at 4.76% in LAB+BS10.0 (*p* < 0.01), underscoring *B. subtilis*’s role in lipid preservation. *B. subtilis* synergizes with LAB to enhance fiber degradation (decrease NDF) and lipid retention (increase EE), though its dose-dependent effects on WSC and ash warrant further mechanistic exploration.

## Conclusion

The integration of homogeneous LAB consortia with graded doses of *B. subtilis* significantly enhanced the fermentation and nutritional quality of *P. australis* silage. The optimal treatment, LAB-BS10.0 (1 × 10^8^ CFU·kg-1 *B. subtilis*), demonstrated the highest lactic acid-to-total acid ratio (62.3%) alongside marked reductions in NH_3_-N (0.60 ± 0.09 g/kg) and NDF (67.80%). Concurrently, EE increased to 4.76%, emphasizing *B. subtilis*’s role in lipid preservation and fiber degradation. These findings validate the synergistic potential of LAB-BS consortia to improve *P. australis* silage quality, offering actionable insights for scaling its silage production as a sustainable feed resource. This study provides a technical framework for optimizing microbial inoculants in *P. australis* silage system, addressing local forage shortages through innovative biomass valorization.

## Data Availability

The raw data supporting the conclusions of this article will be made available by the authors, without undue reservation.
